# The role of nasal trigeminal nerves expressing TRP channels in modulation of cough threshold and urge to cough – possible clinical application

**DOI:** 10.1186/2045-7022-3-S2-O17

**Published:** 2013-07-16

**Authors:** Jana Plevkova, Zuzana Biringerova, Silvia Gavliakova, Eva Hanuskova, Tomas Buday, Mariana Brozmanova

**Affiliations:** 1 Comenius University, Jessenius Faculty of Medicine, Martin, Slovakia; 2 Comenius University, Jessenius Faculty of Medicine, Department of Pathophysiology, Martin, Slovakia

## 

Cough is a phenomenon frequently associated with upper airway diseases and as a reflex is modulated by many afferent inputs either from respiratory tussigenic areas, but also by afferent drive from other organs. Modulation of cough by nasal afferent inputs could either facilitate cough response or inhibit it in animal models, depending on the type of trigeminal afferents which are stimulated. In recent study we focused on afferents expressing TRPA1, TRPM8 & TRPV3 channels -- channels known as relevant for airway irritants (TRPA1), menthol and other cooling substances (TRPM8) and thymol (TRPV3). Particularly menthol and thymol are substances which are frequently used in over-the-counter medication for cough and common cold based on empirical approach. Objective evidence regarding the modulation of cough in humans has never been reported. 60 human healthy volunteers participated in the study, and they have been challenged by intranasal drops containing agonists of selected ion channels: isocyanate (AITC) & cinnamaldehyde for TRPA1, (-) menthol and (+) menthol for TRPM8 and thymol for TRPV3 ion channels in randomized order (all 10-3 M). Nasal symptom score, cough threshold (C2), urge to cough (Cu) and cumulative cough response had been assessed using capsaicin cough challenge tests. Nasal challenges of TRPA1 relevant agonists induced considerable nasal symptoms, significantly enhanced urge to cough (p < 0.05) but modulation of C2 and cumulative cough response did not reach significance level. Both TRPM8 agonists and TRPV3 agonist thymol administered to the nose significantly modulated all parameters including C2 (p<0.05), Cu (p <0.01) and cumulative cough response (p < 0.01) documenting strong anti irritating and antitussive potential of menthol isomers and thyme. Nasal afferent drive modulates cough reflex in human healthy volunteers and this knowledge could have clinical application involved in relieving lower airway symptoms in subjects with upper airway diseases. The role of trigeminal afferents, olfactory nerve endings, smell perception process and other supramedullar influences have to be taken into consideration as relevant enough to modulate cough response in humans.

